# The stealth effect from a medicinal chemist perspective: definition and updates

**DOI:** 10.3389/fddev.2025.1564120

**Published:** 2025-04-28

**Authors:** Domenico Fuoco

**Affiliations:** Department of Chemical Engineering, Polytechnique Montréal, Montréal, QC, Canada

**Keywords:** nanoparticles, liposome, IV treatments, pk, PD, nano-drugs, stealth effect, functionalization tools

## Abstract

In recent years, there has been a significant increase in literature on emerging nanotechnologies, including nanoparticles, nanorobots, and exosomes, for various therapeutic applications. Additionally, politically driven research initiatives aimed at accelerating COVID-19 vaccine development have further amplified interest in nanoparticles as drug delivery systems. This article provides a personal perspective on the scientific claims surrounding nanoparticles by: (i) analyzing the historical evolution of their terminology, (ii) reviewing the most cited publications in the field, and (iii) offering a professional assessment to guide the next-generation of medicinal chemists. A key aspect of this discussion is the stealth effect, which refers to the ability of nanoparticles to evade recognition and clearance by the immune system, thereby prolonging their circulation time in the bloodstream. This property is essential for enhancing the efficacy of nanoparticle-based therapeutics by improving bioavailability and ensuring targeted drug delivery to diseased tissues. Furthermore, the continuing improvement in ligand-molecules and other functional tools have developed novel strategies and brand-new definition of delivery systems, such as Trojan Horse and Nanorobots.

## Background and rationale

The primary goal of nanoparticle-based drug delivery systems has been to enhance pharmaceutical technology by improving the solubility and bioavailability of hydrophobic drugs while overcoming biological barriers. A logical progression of this technology, following safety and efficacy assessments, is the ability to selectively target specific sites or diseases (Zhuo et al., 2024).

In other words, the main objective is to increase the bioavailability of active pharmaceutical ingredients (APIs) that exhibit poor solubility in the bloodstream, ensuring their precise release at biological targets (Luo, 2020). Hydrophobic drugs, by definition, have low aqueous solubility due to their chemical and physical properties. Many of these drugs have unique molecular scaffolds derived from natural and exotic sources and can be costly to synthesize. They often possess a combination of high molecular weight, alkaloid-derived structures (characterized by one or more nitrogen atoms in lower oxidation states), and high to very high lipophilicity (log P > 2).

Several hydrophobic drugs have demonstrated success in intravenous (IV) formulations, including doxorubicin, cannabidiol, and vitamin E (Boudovitch et al., 2023). However, for pharmaceutical formulation to be suitable for IV administration, it must be clear and transparent to meet regulatory standards. The complete absence of particulate matter or suspensions is a fundamental requirement (Carr et al., 2021).

Medicinal chemists are well acquainted with the quality control and regulatory requirements that govern pharmaceutical development. Table 1 outlines the various sections of a Drug Master File (DMF), which must be compiled for any material intended for pharmaceutical use. Each chapter contains comprehensive documentation on quality control testing, analytical data, results, and validation procedures, all of which justify proceeding to the subsequent chapter.

**TABLE 1:** List of key points that are involved in the development of commercial launch of a novel nanoparticle for IV treatment.

#	Product development stage	Parameters	Field of innovation
1	Ideation	Un-met medical need	*in vitro* and/or *in silico*
2	Ideation	IP (patent and trademark)	Benchmark
3	Ideation	Literature Analysis	*In vitro*/*ex vivo*
4	Ideation	R&D Lab	Formulation and Packaging
5	Platform For Production	GLP Facility	Feasibility vs. Compounding
6	Regulatory Affairs	Ethic Committee	First Formal Validation
7	Preclinical Studies	Certified Animals Facility	Models’ screening/selection
8	Regulatory Affairs	Clinical Trial Application	Claim for main indication
9	Clinica Trial (Phase I and II)	GCP Facility	Drug characteristic key points
10	Pharmacology (PK)	AUC (Peak and Duration)	Half-Life Time
11	Pharmacology (PD)	Efficacy	Specificity
12	Toxicity Profile (Side Effects)	Selectivity	Exposure and Elimination
13	Immunity Response	Type of reactions	Stealth Effect
14	Clinical Trial Phase III	Hospitals	Real-Data Outcomes
15	Regulatory Affairs	Panel Review	License Issuance
16	Platform For Production	GMP Facility	3 First Batch Manufacture (BMR)
17	Platform For Production	GMP Facility	Stability Studies
18	Regulatory Affairs	Marketed rules	Production and Distribution
19	Platform For Production	GMP Facility	Full Scale Up Production
20	Pharmacovigilance (PV)	Phase IV	Patients and Professional Associations

The approval process for novel nanoparticles is time-consuming and expensive, comparable to the approval pathway for New Chemical Entities (NCEs) (Li et al., 2024). This rigorous process is required for any new molecule intended to be classified as an Active Pharmaceutical Ingredient (API) (Lipsky and Sharp, 2001).

Nanoparticles smaller than 300 nm appear transparent upon visual inspection. However, transparency alone does not confirm safety, as toxic compounds and impurities can also pass this test. Therefore, every IV formulation must undergo stringent sterility testing (Desai, 2012). One of the primary techniques used to ensure sterility is ultrafiltration, although this process can subject nanoparticles—particularly those that are not solid and range between 100 and 300 nm—to physical stress (Pasut, 2019).

Most sterile filters used in IV formulations have pore sizes below 200 nm, making nanoparticles above this threshold ineligible for IV use. However, sterility does not inherently guarantee an absence of immunogenicity. Once in the bloodstream, nanoparticles face a complex biological environment, where their interaction with immune cells determines their fate (Sun, 2024).

To evade immune detection, nanoparticles are often coated with specific materials that render them “stealth,” or effectively invisible to the immune system. This stealth effect reduces immunogenicity and can further enhance nanoparticle function by enabling targeted drug delivery through surface functionalization. Functionalization represents the future of nanoparticle-based medicine, paving the way for *ad personam medicine*, novel vaccines, and genetic therapies (Herdiana et al., 2022).

## Introducing the “stealth effect”

The improved nanoparticle-based bioavailability as drug carriers has revealed itself as a gamechanger (if not a disruptive one) for transdermal and/or oral drug delivery (Wen et al., 2023). But in IV therapies, the requirement for extra delivery systems is not always obvious, since nanoparticles can sometimes have side effects that are worse than those of the active substance itself. This underscores the crucial role of surface coating, which improves the stability of the formulation, reduces its immunogenic potential and enables precise targeting of the delivery system to the area of interest.

The work of Schöttler et al. (2016) is a good example of the role of nanoparticle coating in precise medication administration, and the differences in the pharmacokinetics (PK) and pharmacodynamics (PD) of PEG-coated liposomal doxorubicin versus uncoated nanoparticles. The “stealth effect” is the name given to the phenomenon through which nanoparticles avoid recognition and removal by the immune system, just like a stealth aircraft avoids detection by conventional radar (Boudovitch et al., 2023). Irrespective of the size or velocity of these engineered particles, they are invisible to the immune surveillance mechanisms and hence circulate for a longer period and enhance the delivery of the drug (Caracciolo et al., 2015; Caracciolo, 2013).

Liposome-based carriers are the best among nanoparticle platforms. Their phospholipid structure is like human cell membranes; therefore, they are the most biocompatible and least toxic from the medicinal chemistry viewpoint. This property has resulted in their categorization as bio-mimetic and bio-inspired nanoparticles, all of which are biodegradable and stimulate minimal immune response (Laura-Immordino et al., 2006).

However, the main task of nanoparticles is to transport drugs to the target cells; therefore, after the drugs have been delivered, the nanoparticles must be removed from the circulation. If they do not degrade effectively, they will form aggregates that have a longer half-life, accumulate in tissues and may be toxic due to the products formed. Therefore, optimal nanoparticle design must consider not only the functional output, but also the metabolic clearance (Elhassan Taha et al., 2024).

The type of material chosen for the nanoparticles is crucial in IV applications. From the medicinal chemistry viewpoint, the following order is recommended.

✔ Most preferred: Lipids and carbohydrates (short cycle molecules)
✔Second best: Albumin, short chain glycopeptides and pseudo proteins.
✖ Avoid at all costs: Surfactants, large proteins, and long chain carbohydrates.
⚠ Used only in certain clinical situations with constant supervision: Metallic, organometallic, and inorganic complexation methods.

### A data analysis of the patented materials

A review of nanoparticle related patents filed in the past decade is presented in Table 2 below. From the analysis of the abstract of these patents, the materials employed in the nanoparticle formulations can be classified based on their chemical nature. On this basis, the materials used in nanoparticle formulations can be distinguished as carbon based and non-carbon-based. These two categories can further be divided based on the type of material used in each category.

**TABLE 2:** The State-of-the-Art. Quantitative assessment of “materials” used in filing patents as nanoparticles. Overall data from the last 10 years.

Main classification	Sub-categories	%	Top 5 materials	%	Example and Ref.
Not Carbon-based	Metal	23.7	Alloy and Aluminium Titanium Copper Silver Gold	5.0 2.0 1.8 1.5 1.0	Gold nanoparticles in cancer therapy: Sazgarnia et al. 2013
	Semiconductor	14.4	Silicon Germanium GaN Polysilicon GaAs	8.0 1.3 1.2 0.9 0.9	Quantum-Dot for diagnosis via imaging techniques: Kosaka et al, 2013
	Non-Metal	10.5	Water Oxygen Hydrogen Nitrogen Boron	4.0 3.9 2.4 2.0 1.0	Nanoparticle for photodynamic therapy: Fuoco, 2015a; Fuoco, 2015b; Yi et al., 2018
	Ceramic/Glass	11.3	Silica Metal oxide Titania Alumina Nitride	4.6 2.2 1.2 1.2 1.1	Silica nanoparticle in cancer-therapy: Yang and Yu, 2016
	Phosphors	0.5	Phosphor Phosphor -derivates	2.0 0.5	Black phosphorus: Qiu et al., 2018
Carbon-based	Synthetic Polymer	12.9	Resin Plastic Thermoplastic Copolymer Polyester	3.1 1.0 0.9 0.8 0.6	Copolymer-based nanoparticle as delivery system Agrahani and Agrahari, 2018 Ray et al. (2018)
	Biological Molecules	9.3	Proteins Nucleic Cellulose Peptide Oligonucleotide	0.7 0.5 0.4 0.3 0.3	Albumin-based nanoparticles Lei et al. (2021) Elzoghby et al. (2012)
	Non-polymer	8.4	Alkyl Hydrocarbon Surfactant Ester Alcohol	0.9 0.8 0.7 0.5 0.5	Lipid-based nanoparticles Fuoco et al., 2020; Fuoco, 2012a
	Organo-metallic/Crystalline carbon	7.9	Nanotube Fullerene Diamond Graphite Carbide	7.4 1.0 0.8 0.7 0.6	Carbon-based nanoparticles and nanorobots Zhao et al. (2023) Hosseini et al. (2023) Sukumaran et al. (2025)
	Organosilicons	1.2	Silane Silicone Teos Alkoxysilane Siloxane	0.5 0.4 0.2 0.2 0.1	Silicon-based nanoparticles Chen et al., 2024; Tieu et al. 2019

For instance, non-carbon-based nanoparticles can be classified by the Periodic Table of Elements according to whether the material is a metal, semiconductor, non-metal, ceramic/glass or phosphor. On the other hand, carbon-based nanoparticles can be divided into synthetic polymers, biological molecules, non-polymeric structures, complex organometallic/crystalline materials and organo-silicones (Leitch et al., 2012).

Another interesting result that can be derived from this analysis is that the first 47 subcategories are represented by 71.7% of all the patented nanoparticle materials. Missing or misclassified data may be present, however, a ±10% margin of error suggests that up to 80% of all the nanoparticles that have been patented can be attributed to these subcategories. This means that about 20% of the possible chemical families have not been explored for nanoparticle applications (Castagnolo, 2023).

One of the most fascinating aspects of chemistry is the possibility to vary the molecular structures during bio-isosteric substitutions. The synthesis of new subcategories of bio-mimetic, non-synthetic molecules is possible through replacement of certain atoms or functional groups leading to further innovation in nanoparticle design (Wanieck et al., 2017).

### The utilization of the database as a source of information

Databases are typically employed as information sources that confirm the current state of the research rather than contribute to the creation of new knowledge. At first glance, it may seem counterintuitive to consider a database–which is a collection of already published information–as a source of innovation. However, in the field of medicinal chemistry, it is necessary to ask not ‘why?’ but ‘why not?’ (Fuoco, 2012b).

Some of the most important databases that a researcher in the pharmaceutical field should consult before reaching a conclusion or formulating a new research question are presented in Table 3 (Murcko, 2018).

**TABLE 3:** Reality vs. Fiction. Data-driven analysis of nanoparticles usage in clinic.

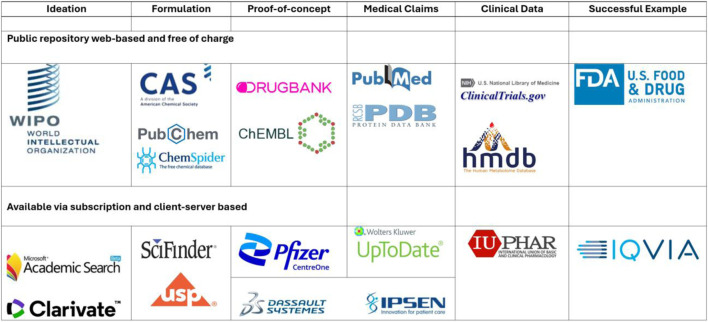

Most of these key databases are public repositories of information including PubChem, PubMed, ClinicalTrials.gov, and DrugBank. These resources provide open access to massive data on chemical properties, clinical trials and drug formulations. But professional associations like the American Chemical Society (ACS) and the American College of Clinical Pharmacology (ACCP) hold private databases with very specific and focused datasets. These databases are often proprietary chemical property data and scientific insights that are curated and made available to paid subscribers only (Cao et al., 2024).

In addition to the public and academic repositories, industry specific databases are also very important in pharmaceutical development. Each of these resources is useful in building a Drug Master File (DMF), a single comprehensive regulatory document that is required for the approval of pharmaceutical materials by regulating bodies like the US Food and Drug Administration (FDA) (Sravanti et al., 2021).

## Bibliometrics: google trends vs. clarivate vs. FDA

At the product development stage, it is difficult to make a professional judgment about the feasibility of a new technology just from the literature. This is especially the case in the idea generation phase when there may be little to no official information and the results of the experiments may not be very reliable or supported by other work.

One interesting example of this problem is the divide between the public and scientific communities. Some words, for instance Nanosome™ and nanorobots, are often used in searches and trademarks of approved medical products and have no real application in them. This can be compared to the debate on extraterrestrial life–popular, but still based on assumptions (Debus et al., 2019).

This further complicates the model because not all information sources are created equal. For example, Google Scholar, one of the most popular public repositories, is not highly regarded by researchers, while Clarivate, one of the most cited academic databases, is only available through expensive subscriptions (Fuoco, 2012a). This difference can be attributed to the difference between open access and closed access knowledge in research.

Circling back to nanotechnology, several well-known scientists have published papers on the possible clinical implications of nanorobots. This is one of the most drastic applications of nanotechnology, but up to now, the U.S. Food and Drug Administration (FDA) has not approved any nanorobots for clinical trials or therapeutic use (Uzundurukan et al., 2024). This shows that, while scientific hype may result in theoretical progress, it does not necessarily lead to real medical applications.

### Regulatory and manufacturing challenges in nanoparticle development

Beyond the chemical and pharmaceutical aspects of nanoparticle development, two key non-chemistry factors are what determine the success of a nanoparticle-based therapy: 1) a regulatory approval process whereby nanoparticles are evaluated rigorously by regulatory agencies like the U.S. Food and Drug Administration (FDA), the European Medicines Agency (EMA), and Health Canada for their commercialization. An important part of this approval process is the submission and review of a Drug Master File (DMF) that contains extensive information on quality, safety, and efficacy. Regulatory approval is needed to guarantee that the product complies with the established pharmaceutical standards and gain authorization for market release. 2) The manufacturing scale-up and technology transfer which involves the transition from laboratory scale research to large scale commercial production is ruled by strict Good Laboratory Practice (GLP) and Good Manufacturing Practice (GMP) guidelines. This includes the transfer of batch manufacturing records (BMR) from small scale experimental formulations to full scale industrial production, to guarantee product consistency, sterility, and regulatory compliance. These two factors–regulatory validation and large-scale production feasibility–are as crucial as the scientific innovation itself. Without them, even the most promising nanoparticle formulations cannot progress beyond the research phase and into clinical and commercial applications (Isibor, 2024).

### Basic concepts in nanoparticle production

#### Stealth effect

The stealth effect in nanoparticles is the phenomenon of nanoparticles avoiding recognition and being rapidly cleared from the immune system. This effect is important for improving the delivery of drugs because it increases the availability of nanoparticles at the target tissues. This stealth effect is achieved using surface coatings such as PEG (Boudovitch et al., 2023).

#### Functionalization tools

Nanoparticle functionalization is the process of altering the surface of nanoparticles to make them more stable, bioavailable and targeted. Different strategies enable the nanoparticles to attach to the diseased tissues more specifically, thus enhancing the therapeutic impact. The variety of materials used for functionalization is extensive and encompasses lipids, proteins, carbohydrates, and synthetic polymers. Since the 1990s, the FDA has cleared more than 50 nanotechnology products for clinical tests across four classes of pharmaceuticals. Despite this, there are only two of these technologies that have received FDA approval for clinical use, including a license to market with an NDC number. All pharmaceutical products sold in pharmacies must display the NDC number on the label (Caracciolo, 2013; Mitchell et al., 2021).

#### Exosomes

Exosomes have been viewed as one of the major new technologies of the last decade. A Nature article titled “Exosomes: key players in cancer and potential therapeutic strategy” by Dai et al. (2020) reviews the subject. As a Lead Auditor in Pharmaceutical Quality Management, it is important to correct the misconception that exosomes are an emerging technology and have not been approved by the FDA for therapeutic use in cancer (Zhang et al., 2020). Exosomes, as defined by RM Johnstone in 1987, are extracellular vesicles which are phospholipid bilayer membrane from the same cellular membrane that releases them (Johnstone et al., 1987). Nevertheless, the term was already in use in the early 1970s to describe genetic material transferred between individuals, to distinguish these “extra genes” from episomes—DNA fragments located near the host’s chromatin structure (Mishra and Tatum, 1973).

#### Trojan-horse strategy

Physiological phenomena are often explained by pharmacologists using historical analogies, viewing the host-external perturbing factors interaction as a strategic battle between host and xenobiotics, toxins, viruses, and bacteria (Hermosillo-Abundis et al., 2024). The Stealth Effect in nanoparticle-based drug delivery systems is similar in that it allows therapeutic agents to penetrate the host system without eliciting an immune response. The Trojan Horse Strategy applies this principle to allow nanoparticles to pass through cellular defenses, to target intracellular receptors or to directly modify the host genome to influence cellular functions to support the intended therapy (Patra et al., 2018).

#### Nanorobots

Nanorobots in nanomedicine were first proposed by R. A. Freitas in the early 2000s in his paper “Applications of Nanorobotics to Dentistry” (Freitas Jr, 2000). Dental nanorobots of the micrometer scale which he proposed were supposed to be capable of exact navigation through human tissues, acquiring energy, sensing and manipulating their environment, and achieving cytopenetration without harming the cellular structure. Nanorobots as nanomedicine are still in the future, 25 years after Freitas’s proposal which was likely inspired by Kubrick’s 2001: A Space Odyssey, and more than 1,400 related articles published in PubMed (Mao et al., 2025). Nanorobotics is a field that has been researched extensively but no such technology has been approved for clinical trials.

Key concepts: summary table

**Table:** 

Technology	Composition	Functional mechanism	Existing techniques
Stealth Effect in Nanoparticles	Nanoparticles coated with PEG, lipids, or polymers	Prevents immune system recognition, prolonging circulation time and increasing drug availability at target sites	PEGylation, lipid coatings, polymer-based shielding (Boudovitch et al., 2023)
Nanoparticle Functionalization	Lipids, proteins, carbohydrates, synthetic polymers	Enhances nanoparticle stability, bioavailability, and targeted delivery to diseased tissues	Ligand-functionalized nanoparticles, antibody-conjugated nanoparticles (Caracciolo, 2013 Mitchell et al. (2021)
Exosomes	Extracellular vesicles (phospholipid bilayer)	Facilitates intercellular communication, potential for drug delivery	Isolation by ultracentrifugation, size-exclusion chromatography, microfluidics (Johnstone et al., 1987)
Trojan Horse Strategy	Nanoparticles, exosome-like vesicles, viral vectors	Enables nanoparticles to bypass cellular defenses, target intracellular receptors, or modify host genome	Ligand-targeted nanoparticles, RNA/DNA delivery systems, exosome-mimicking carriers (Patra et al., 2018)
Nanorobots in Nanomedicine	Theoretical—micrometer-scale robotic devices	Predicted to navigate tissues, interact with cellular components, and achieve cyto-penetration without damaging cells	Research-stage only; concepts include DNA origami robots, magnetically controlled microrobots (Freitas Jr, 2000; Mao et al., 2025)

## Conclusion

The main purpose of this paper is to provide a practical guideline (*vade mecum)* for the medicinal chemist on how to tell the difference between the scientific feasibility of using nanoparticles as an oral drug delivery system and the feasibility of such use in the real world. In this way, this article provides a structured, data-driven analysis and thus forms a rational basis for designing nanoparticles that are efficient from the functional point of view and compatible with the regulatory system.

The pharmaceutical industry is completely different from consumer markets where marketing is the only factor that determines the success of a product. By contrast, the development of drugs is strictly supervised, which makes it very difficult to replicate success after a product has been approved. This is because every project needs to be strongly validated from the time it is thought of, up to the time it is implemented clinically.

Two non-chemical factors which are most important for the success of nanoparticle-based therapies are.

• Regulatory Approval–The approval of major health agencies including FDA, EMA and Health Canada is a function of safety and efficacy which in turn requires Drug Master File (DMF) submission.
• Manufacturing Scale-Up–The move from the laboratory to industrial scale production must meet the standards of Good Laboratory Practice (GLP) and Good Manufacturing Practice (GMP) to guarantee the product’s quality, sterility and compliance.

### Key takeaways

This review also identifies the crucial advances and the main difficulties in the field of nanoparticle-based drug delivery.

- Stealth effect: The ability of nanoparticles to evade immune detection is paramount for enhancing bioavailability and prolonging circulation time (Boudovitch et al., 2023; Caracciolo et al., 2015).
- Functionalization and targeting: The development of surface modifications allows for a precise delivery of drugs to the target tissues, which is a significant step towards the realization of the concept of personalized medicine (Caracciolo, 2013; Mitchell et al., 2021).
- Data driven innovation: The patent analysis shows though much remains to be achieved, about 20% of the nanoparticle material options are still unexplored and can be used further (Castagnolo, 2023).
- Regulatory and manufacturing barriers: Even the most promising nanoparticle formulations are difficult to translate to clinical practice due to the stringent regulatory standards and the problems in scaling up the production (Elhassan Taha et al., 2024).
- Hype and reality of nanorobots: Currently, there are no nanorobots in clinical practice, which proves that the scientific ideas are still far from their practical implementation (Freitas Jr, 2020; Uzundurukan et al., 2024).

The use of nanoparticles for drug delivery is an evolving field, and although much remains to be achieved before moving from concept to practice, it is imperative that scientific innovation is accompanied by regulatory adaptation and large-scale manufacturability. In the process of the development, the cooperation between chemists, biologists, engineers and regulatory bodies will be required to realize the potential of the concepts in practice and provide the much-needed solutions in the medical field.

## Data Availability

The original contributions presented in the study are included in the article/supplementary material, further inquiries can be directed to the corresponding author.
