# Supplementary material for: The stealth effect from a medicinal chemist perspective: definition and updates
Source: Front Drug Deliv. 2025 Apr 28;5:1564120. doi: 10.3389/fddev.2025.1564120 (PMC12360436; doi:10.3389/fddev.2025.1564120)
Supplement: Supplementary file 1 [file Supplementaryfile1.pdf]

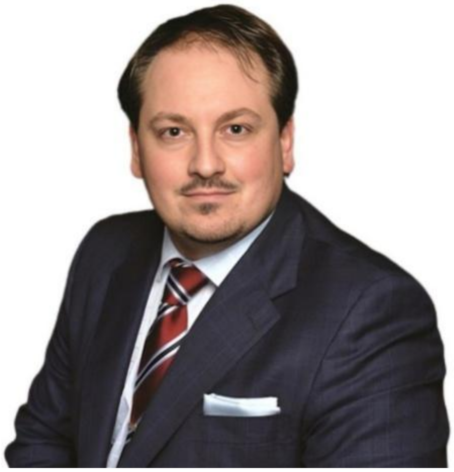

Domenico Fuoco, *PhD, PharmD, Chemist*

Associate Professor
Department of Chemical Engineering
École Polytechnique, Montréal

Areas of expertise
Biodiversity| Sustainability pathways| Characterization of nanostructure | Analytical chemistry | Semi-solid formulation | Supply chain | Molecular modeling | Pharmaceutical technology | Regulatory Affairs and GMP Compliance

Selected Achievement
7 Patents: 3 Granted USPTO/WIPO + 4 Applications PCT
40 Scientific communications in conference outside Canada in the last 10 years.
500 products (NHP OTC, Rx, C/T, N) launched to the market worldwide since 2006

2025 - Lead Auditor ISO 19011:2018 EU GMP / US FDA CFR 211
2024 - Executive Vice President, Biovantek – Labomar Canada
2023 - Senior Regulatory Affairs Officer, Medelys International Labs
2022 - Health Canada – Narcotic Exemption for Psilocybin – Award
2021 - SVP Innovation and Compliance at CYBIN™, Public company
2020 - Co-Chair ISCRE – Chemical Reaction Engineering - Award
2019 - CQIB Grant – Biotech Start-Up of the Year - Award
2018 - Chief Innovation Officer at WeedMD™, Public company
2017 - Head of Science – Earth Technology and Science Trust™ - Award
2015 - Henry Shibata Fellowship, Cedar Cancer Foundation - Award
2010 - International Year of Biodiversity™ Award, Colombia – Award

Dr. Domenico Fuoco is a certified lead auditor for ISO 19011:2018, with over 100 audits conducted in Pharmaceutical Quality Management Systems across the European Union, Turkey, and North America. Since the early 2000s, he has been directly involved in product and process development finalized to the licensing and market approval of over than 500 pharmaceutical products worldwide.

In late 2022, Dr. Fuoco returned to academia as an Associate Professor, bringing extensive industry expertise to his research and teaching. Unlike traditional academics, his authority in the field is not solely based on peer-reviewed publications, but on his 24 years of experience in the pharmaceutical industry, where he has contributed to hundreds of medical claims and patents.

Dr. Fuoco unique background bridges the gap between scientific innovation and regulatory application, making him a key figure in the translational development of pharmaceutical technologies.
